# ATP Regeneration from Pyruvate in the PURE System

**DOI:** 10.1021/acssynbio.4c00697

**Published:** 2025-01-04

**Authors:** Surendra Yadav, Alexander J. P. Perkins, Sahan B. W. Liyanagedera, Anthony Bougas, Nadanai Laohakunakorn

**Affiliations:** Centre for Engineering Biology, Institute of Quantitative Biology, Biochemistry and Biotechnology, School of Biological Sciences, University of Edinburgh, Edinburgh EH9 3FF, U.K.

**Keywords:** cell-free protein synthesis, synthetic cells, synthetic biology, PURE, ATP regeneration, synthetic metabolism

## Abstract

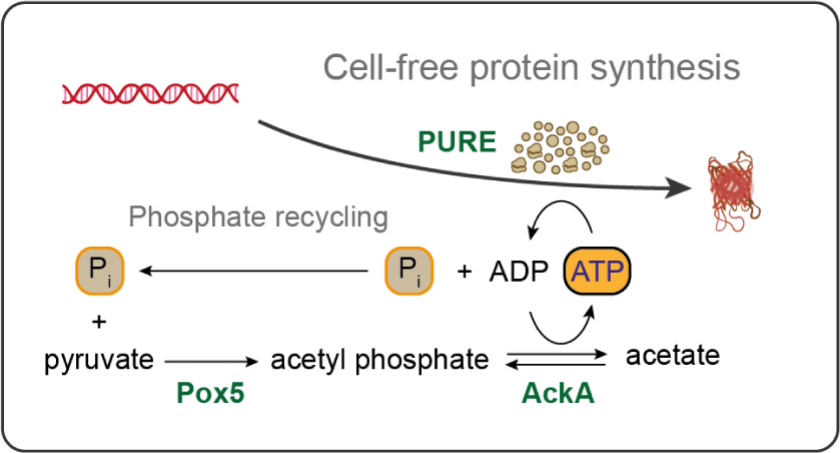

The “Protein synthesis Using Recombinant Elements” (“PURE”) system is a minimal biochemical system capable of carrying out cell-free protein synthesis using defined enzymatic components. This study extends PURE by integrating an ATP regeneration system based on pyruvate oxidase, acetate kinase, and catalase. The new pathway generates acetyl phosphate from pyruvate, phosphate, and oxygen, which is used to rephosphorylate ATP *in situ*. Successful ATP regeneration requires a high initial concentration of ∼10 mM phosphate buffer, which surprisingly does not affect the protein synthesis activity of PURE. The pathway can function independently or in combination with the existing creatine-based system in PURE; the combined system produces up to 233 μg/mL of mCherry, an enhancement of 78% compared to using the creatine system alone. The results are reproducible across multiple batches of homemade PURE and importantly also generalize to commercial systems such as PURExpress from New England Biolabs. These results demonstrate a rational bottom-up approach to engineering PURE, paving the way for applications in cell-free synthetic biology and synthetic cell construction.

## 1 Introduction

Cell-free protein synthesis (CFPS) harnesses the core biological processes of transcription and translation to generate mRNA and protein from a DNA template, in controlled biochemical reactions outside of living cells. These systems were originally used to elucidate fundamental mechanisms in molecular biology, but recently have been deployed for a wide range of applications within synthetic biology and biotechnology.^1^ These include practical applications such as biomanufacturing, biosensing, and diagnostics,^2^ as well as more fundamental research involving bottom-up construction of biomolecular systems which aim to mimic aspects of living cells.^1^

CFPS reactions are composed of three categories of components: 1) a DNA template encoding for a gene of interest; 2) small-molecule substrates and cofactors such as nucleoside triphosphates (NTPs), amino acids, and tRNA; and 3) the molecular machinery needed to carry out *in vitro* protein synthesis, which includes enzymes, translation factors, and ribosomes.

This molecular machinery is supplied either in the form of clarified cell lysates, which can be obtained from a number of different organisms;^3,4^ or they can be recombinantly produced and individually purified. Such a defined system, known as PURE (Protein synthesis Using Recombinant Elements) was originally developed by Shimizu and coworkers in 2001,^5^ and consists of purified *Escherichia coli* ribosomes and 36 protein factors, which together carry out transcription, translation, tRNA aminoacylation, and biochemical energy regeneration, the four processes needed to sustain cell-free protein synthesis.

Since the PURE system is defined, it has been used in a number of studies which can take advantage of this, such as unnatural amino acid incorporation and *in vitro* directed evolution.^6^ It is also an ideal starting point for the construction of more complex subsystems which may eventually be combined into a replicating, autonomous synthetic cell.^7−9^ However, these ambitious applications are challenged by the fact that the system in its current form is limited in both protein synthesis yield (for example, ∼ 200 μg/mL green fluorescent protein, GFP) and reaction lifetime (∼1 h).^10^ The limitations of PURE include low processivity and speed of translation leading to truncation, inactive products, and stalled ribosomes;^11,12^ protein misfolding;^13^ tRNA and translation factor depletion; and accumulation of inhibitory misfolded mRNA^14^ and inorganic phosphate.^12^

To an extent these limitations are shared with the more intensively studied lysate-based systems. Early work in the 2000s focused on engineering *E. coli* lysates to improve the performance of batch-mode CFPS reactions, and a number of limitations were discovered and addressed.^15^ It was recognized that a major limitation was the buildup of inorganic phosphate (P_i_) as the reaction proceeds, resulting from ATP hydrolysis; this phosphate chelates and reduces the concentration of free Mg^2+^ ions in the system. Magnesium is a cofactor for many enzymes involved in protein synthesis, and additionally stabilizes the structure of the ribosome,^16^ and so a reduction in free Mg^2+^ due to phosphate accumulation was proposed as a cause of batch reaction termination after only ∼1 h.^17^

Since CFPS is a very energy intensive process, consuming ∼4–5 ATP equivalents per peptide bond synthesized (BioNumbers BNID 107782),^18^ its biochemical energy must be replenished to allow the reaction to proceed beyond a few minutes *in vitro*. Simple ATP regeneration schemes involving substrate-level phosphorylation, using enzyme/substrate pairs such as pyruvate kinase/phosphenolpyruvate,^19^ acetate kinase/acetyl phosphate,^20^ or creatine kinase/creatine phosphate,^21^ result in a unidirectional transfer of phosphate to ATP, which then accumulates following ATP hydrolysis.

A solution to this problem is to either remove the accumulated phosphate through physical means (e.g., continuous exchange^22,23^ or flow reactors,^24,25^) or alternatively to engineer a biochemical regeneration scheme which recycles phosphate. The first such demonstration was by Kim and Swartz who supplemented *E. coli* lysates with pyruvate oxidase from *Pediococcus* sp.^26^ Pyruvate oxidase catalyzes the condensation of pyruvate and inorganic phosphate to produce acetyl phosphate, which regenerates ATP through its conversion to acetate; the second reaction is catalyzed using endogenous acetate kinase already present in the extract. This system produces the phosphorylated substrate acetyl phosphate *in situ*, while avoiding phosphate accumulation, and the authors showed that both the reaction yield and lifetime were increased. Current high-yield systems also benefit from phosphate recycling, such as the maltose/maltodextrin-based “TXTL” systems developed by Noireaux and coworkers which condense maltose with phosphate to form glucose-1-phosphate, which is subsequently fed into the glycolytic pathway to regenerate ATP. Such systems are able to achieve up to 4 mg/mL of GFP, with reaction lifetimes up to 20 h.^27,28^

**Figure 1:**
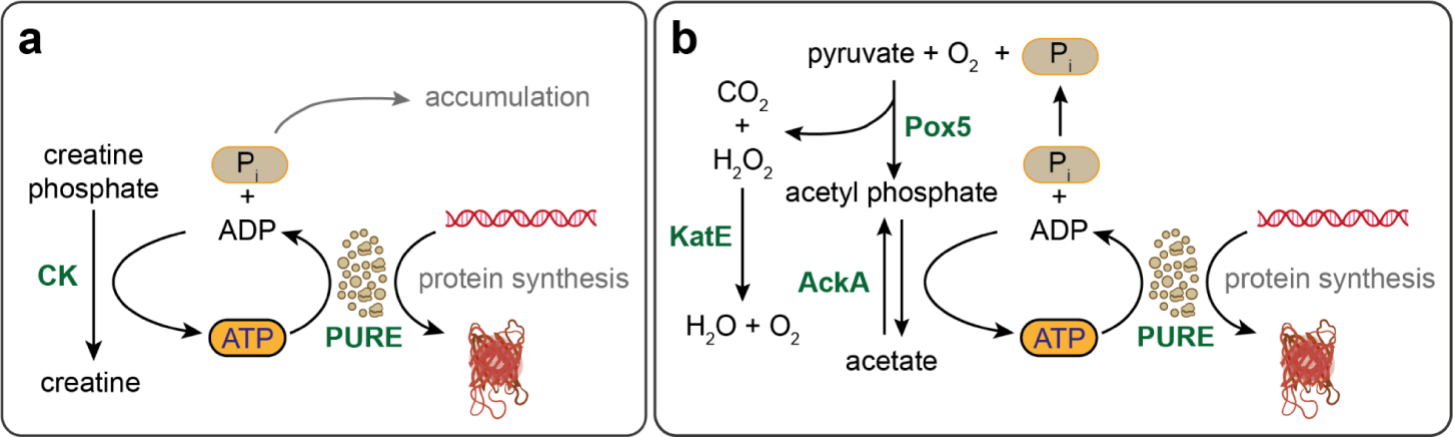
Schematic of CP/CK (a) and PAP (b) as ATP regeneration pathways in the PURE system. (a) CP/CK employs creatine phosphate as a substrate to regenerate ATP from ADP, utilizing the enzyme creatine kinase (CK). This process results in the accumulation of inorganic phosphate (P_*i*_) as a byproduct. (b) PAP utilizes pyruvate, inorganic phosphate, and molecular oxygen as substrates to regenerate ATP, mediated by the enzymes pyruvate oxidase (Pox5) and acetate kinase (AckA). Additionally, the enzyme catalase (KatE) is involved in the breakdown of hydrogen peroxide, a byproduct of the Pox5 reaction, into water and molecular oxygen.

Unlike lysates, PURE systems typically use a creatine phosphate/creatine kinase (CP/CK) energy regeneration scheme, and hence are proposed to suffer from phosphate accumulation^12^ (Figure 1a). We hypothesized that implementing a similar phosphate recycling scheme to that developed by Kim and Swartz should directly address the limitations of reaction yield and lifetime in PURE (Figure 1b).

In this work we demonstrate the production and purification of three enzymes: *Lactobacillus plantarum* pyruvate oxidase (Pox5), *E. coli* acetate kinase (AckA), and *E. coli* monofunctional catalase (KatE). We supplement the enzymes into a PURE system produced in-house, and demonstrate that the new “pyruvate-acetate pathway” (“PAP”) can energize the PURE system using pyruvate. We apply a design of experiments approach to rationally improve the performance of the pathway, and find that the optimized pathway can power the synthesis of 72 μg/mL of fluorescent protein mCherry over 4 h. Compared to the existing creatine phosphate/creatine kinase system, which produces ∼130 μg/mL, this pathway is not an effective replacement. However, the two pathways in combination can produce up to 230 μg/mL of mCherry after 4 h. Importantly, the results generalize when the pathway is supplemented into commercial PURExpress systems (New England Biolabs).

This work demonstrates that alternative energy regeneration schemes can be straightforwardly implemented in combination with the PURE system. These results as well as other similar studies combining PURE with synthetic metabolic systems^29−31^ pave the way for rationally improving the PURE system’s performance through bottom-up construction.

## 2 Results and Discussion

### 2.1 *L. plantarum* Pyruvate Oxidase Can Be Recombinantly Expressed in *E. coli*

Genes (*pox5* from *L. plantarum*, codon optimized for *E. coli* using the IDT Codon Optimization Tool; *ackA* and *katE* from *E. coli*) encoding the three pathway enzymes were obtained as linear DNA fragments (gBlocks, IDT) and individually cloned into the pET21a expression vector. A 6x-His tag was incorporated at the C-terminus of each enzyme to facilitate affinity purification.^32^ The enzymes AckA and KatE were then overexpressed in BL21(DE3) cells using conventional IPTG induction at 37 °C for 3 h.^33^ However, during the heterologous expression of Pox5 in *E. coli* under similar conditions, the protein was found exclusively in the insoluble fraction of the cellular lysate (Figure S1). To address this, we implemented a modified overexpression protocol by lowering the expression temperature to 15 °C and extending the induction period to 15 h.^34^ This adjustment successfully facilitated the extraction of a fraction of the enzyme within the soluble lysate (Figure S1). After protein production, the enzymes were purified using Ni-NTA affinity chromatography,^32^ and their activities were verified through individual enzymatic assays (Figures S2–S4).

### 2.2 Phosphate Buffer Activates PAP without Inhibiting Protein Synthesis in PURE

Initially, we verified the thermodynamic and kinetic feasibility of PAP (Figure S5, Tables S1 and S2). We then proceeded to test the pathway by adding the following components into a CFPS reaction: the pathway enzymes (Pox5, AckA, and KatE); two cofactors for Pox5, thiamine pyrophosphate (TPP) and flavin adenine dinucleotide (FAD); the PURE system; pyruvate; and a modified energy solution. In order to ensure energy regeneration occurs solely via PAP and not the existing CP/CK system, we omitted creatine phosphate from the energy solution formulation (we verified that the alternative approach, of removing the creatine kinase enzyme from the PURE system, also gave the same results, Figure S6). Phosphate was not added as a substrate initially as we expected the pathway to utilize the inorganic phosphate generated from cell-free protein synthesis through the consumption of ATP. Finally, we replaced magnesium acetate with magnesium glutamate, in order to promote the ATP-producing direction of the AckA reaction.

**Figure 2:**
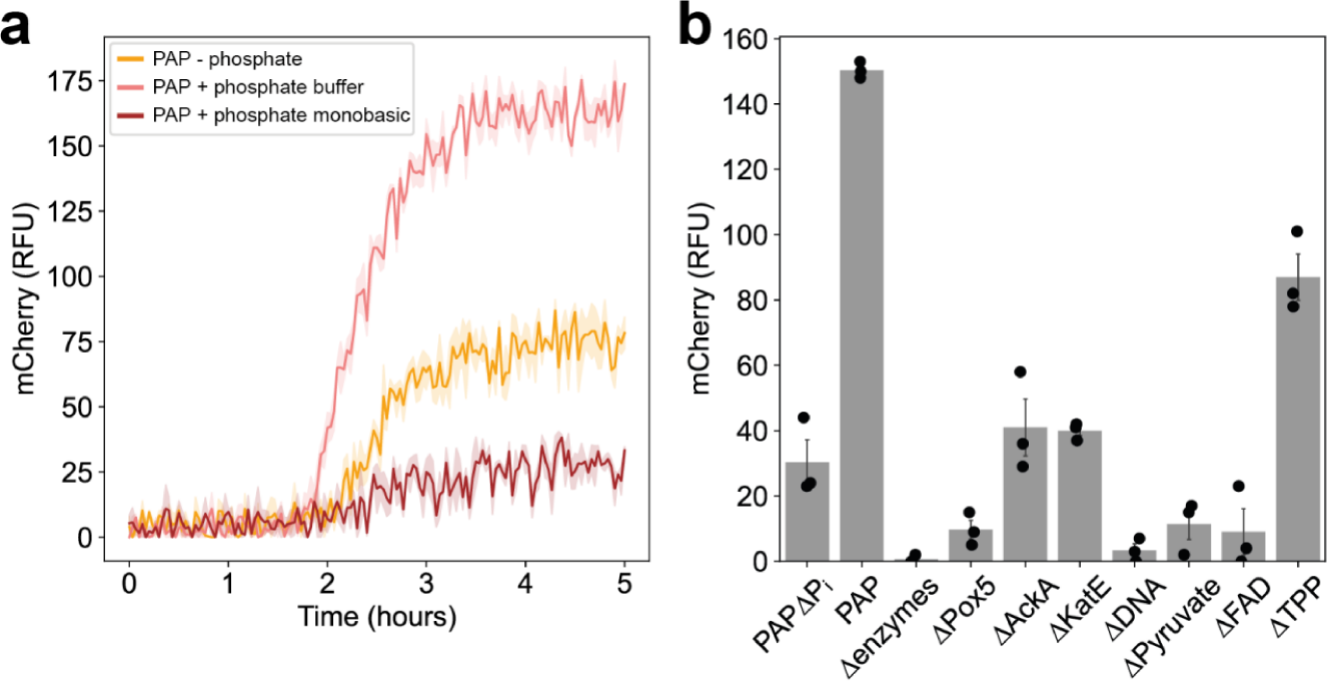
(a) PAP functioned effectively as an ATP regeneration pathway in the PURE system when it was supplemented with 10 mM exogenous phosphate in the form of a potassium phosphate buffer at pH 7. In contrast, 10 mM monobasic potassium phosphate inhibited the reactions. (b) Final protein synthesis yields from PAP-powered reactions after 5 h are reported. Protein synthesis did not occur in the absence of the mCherry DNA template (ΔDNA) or the pathway enzymes (Δenzymes). Pyruvate, Pox5, and FAD are critically important for the pathway’s function, while the pathway retained some activity with the exclusion of phosphate, AckA, and KatE. The Pox5 cofactor TPP is the least essential component, indicating that some TPP might remain bound to the enzyme after purification. The final yield of the PURE + PAP system of 150 ± 1 RFU corresponded to 46.5 ± 0.5 μg/mL of fluorescent mCherry. All experiments were performed in triplicate. Data are shown as mean ± s.e. (*n* = 3).

This approach initially led to markedly low mCherry protein yields (∼8% of the yield from the CP/CK system) (Figure 2a). We hypothesized that the diminished protein yield stemmed from low levels of inorganic phosphate within the reaction, which limits the substrate availability for the Pox5 enzyme.^35^ To address this, we tested the addition of two different phosphate sources in the reaction, monobasic potassium phosphate (KH_2_PO_4_, pH 4.5) and potassium phosphate buffer (a mixture of monobasic and dibasic potassium phosphate K_2_HPO_4_, pH 7). Remarkably, supplementing with 10 mM potassium phosphate buffer significantly increased CFPS output, whereas supplementation with monobasic potassium phosphate inhibited protein synthesis (Figure 2a).

To verify this effect and further characterize these observations, we carried out a titration of the two phosphate sources in PURE reactions using the original CP/CK energy regeneration system. We observed that the addition of monobasic potassium phosphate at concentrations ≥15 mM inhibited protein synthesis, whereas the supplementation of phosphate buffer up to 20 mM had no inhibitory impact on the reactions (Figures S7 and S8).

We next carried out experiments using PAP + PURE with the exclusion of various components (Figures 2b and S9). We observed that the exclusion of pathway enzymes and DNA effectively abolished protein synthesis activity. Similarly, the exclusion of pyruvate, Pox5 or FAD led to low levels of protein synthesis. However, we observed that the exclusion of TPP from PAP still resulted in a system with significant protein synthesis activity. This finding is consistent with the hypothesis that TPP binds tightly to the Pox5 enzyme, and a fraction of TPP remains bound to the enzyme even after the protein purification process is completed,^35^ resulting in the enzyme being partially active in the absence of exogenously added TPP.

The KatE enzyme was found to be crucial for the efficient functioning of the pathway, as its exclusion resulted in a significant decrease in protein yields (Figure 2b). The absence of KatE may lead to the accumulation of hydrogen peroxide within the reaction, which can directly oxidize protein thiol groups, particularly cysteine residues, and initiate further radical-mediated damage. This oxidation process can cause protein dysfunction, misfolding, and aggregation, ultimately resulting in a loss of protein synthesis activity.^36,37^ We verified the inhibitory effect of hydrogen peroxide on protein synthesis (Figure S10) and found an inhibition constant of ∼5 mM; since H_2_O_2_ buildup is proportional to pyruvate depletion, we expect potential accumulation of up to ∼20–30 mM H_2_O_2_ thus corroborating the need for KatE.

Furthermore, we observed that protein synthesis activity persisted at a low level even when the AckA enzyme, the sole enzyme responsible for ATP regeneration in the system, was excluded (Figure 2b). We hypothesize that this residual activity may be attributed to trace amounts of copurified AckA present within the PURE protein mixture. PURE systems produced using the affinity chromatography method are susceptible to contamination: Lavickova et al. report that between 5–12% of the total purified protein content in OnePot PURE is composed of non-PURE proteins,^10^ and likewise Villarreal et al. report between 4–11% in the TraMOS system.^38^ As AckA is present in high abundance in *E. coli*,^39^ we suspect that its contamination explains the residual ATP regeneration from acetyl phosphate.

With the supplementation of 10 mM potassium phosphate buffer, PURE reactions using the PAP energy regeneration scheme achieved a final protein yield of 46.5 ± 0.5 μg/mL, a 5-fold increase compared to phosphate-free reactions (Figure 2b). Encouraged by these enhancements, we decided to adopt a design of experiments (DOE) approach for pathway optimization, with the aim of further increasing protein yields using PAP.

### 2.3 PAP Can Be Optimized Using a Design of Experiments (DOE) Approach

**Figure 3:**
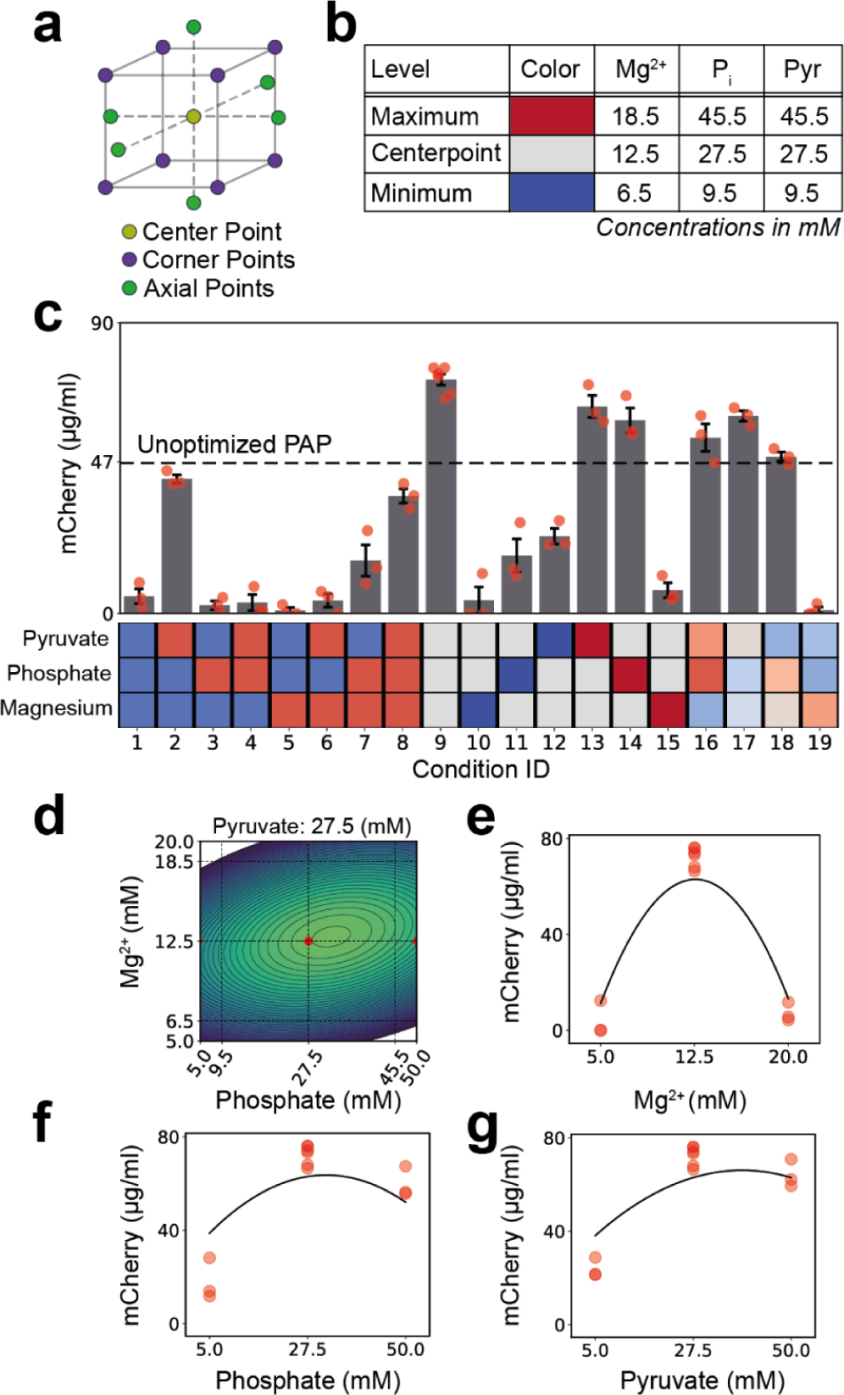
Optimization of PAP using a design of experiments approach. (a) Illustration of the geometric relationship between the different elements within the circumscribed central composite design (cCCD). (b) Table of the design space boundaries with the maximum, minimum, and centerpoint concentrations for each factor. (c) Mean and standard error of the mCherry yield after 5 h, for each condition tested. Individual data points are plotted as red markers, and the assigned levels for each condition are visualized in the heat map below. The mCherry yield of the unoptimized system is plotted as a dashed line at 46.5 μg/mL. (d) Contour plot of the model predicted yield between magnesium and phosphate, where pyruvate is fixed at 27.5 mM. Experimental data points are plotted as red markers with size scaled by yield. (e–g) Plots of the mCherry protein yield as a function of each factor, with the others fixed at their centerpoint levels: experimental data are indicated with red markers, and the model predictions with the solid line.

To enhance the performance of the pathway, we first adjusted the initial concentrations of key components pyruvate, phosphate buffer, and magnesium glutamate, using a rational design of experiments (DOE) approach. We utilized a circumscribed central-composite design (cCCD) to efficiently explore the design space and examine the interactions between these factors (Figure 3a).^40^ This choice was informed by previous findings that demonstrated a nonlinear relationship between Mg^2+^ and protein yield in CFPS reactions, as well as the known interaction between Mg^2+^ and phosphate.^41^ The experimental design space was defined by setting maximum and minimum levels for each factor based on plausible ranges encountered in the literature (Figure 3b).^12,26,42,43^

Initial examination of the data (Figures 3c and S11, Table S3) showed that six conditions had an enhanced performance compared to the unoptimized system, with the best performing condition corresponding to the center point (Mg^2+^ = 12.5 mM, phosphate = 27.5 mM, and pyruvate = 27.5 mM). This condition yielded 74.5 ± 0.8 μg/mL of mCherry, an increase over the baseline of 60.2%. Notably, the worst performing conditions corresponded to high or low Mg^2+^ values, highlighting the sensitivity of the reaction to Mg^2+^ concentration.

In order to probe the behavior of the system more quantitatively, we carried out a response surface analysis by fitting an unsaturated linear regression model to the data, which included linear and quadratic terms for each factor, and a single interaction term between Mg^2+^ and phosphate,
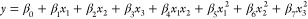
1where the variables *x*_1_, *x*_2_, and *x*_3_ correspond to magnesium, phosphate, and pyruvate concentrations respectively, and *y* the yield of mCherry. The final fitted model parameters are given in Table S4; this yielded *R*^2^ = 0.83 with respect to the cCCD training data, and *R*^2^ = 0.42 on held-out validation data outside the original data set, which suggests a moderate degree of overfitting, although the model predictions are more accurate at higher expression levels (Figure S12). The contributions of model terms to predicting the response can be quantified using a t-statistic (Figure S13),^44^ which shows strong quadratic dependencies for all three substrates, with Mg^2+^ concentration being the most sensitive factor, in agreement with the initial qualitative observations. The interaction term between Mg^2+^ and phosphate is also significant.

The overall response can be visualized in Figure 3d–g, which shows a sharp optimum dominated by Mg^2+^ concentration, with weaker dependencies on pyruvate and phosphate. The interaction between Mg^2+^ and phosphate is illustrated in the contour plot in Figure 3d, which shows that the Mg^2+^ optimum varies weakly as a function of phosphate concentration.

### 2.4 Combining ATP Regeneration from PAP and CP/CK Improves Protein Yield in PURE

**Figure 4:**
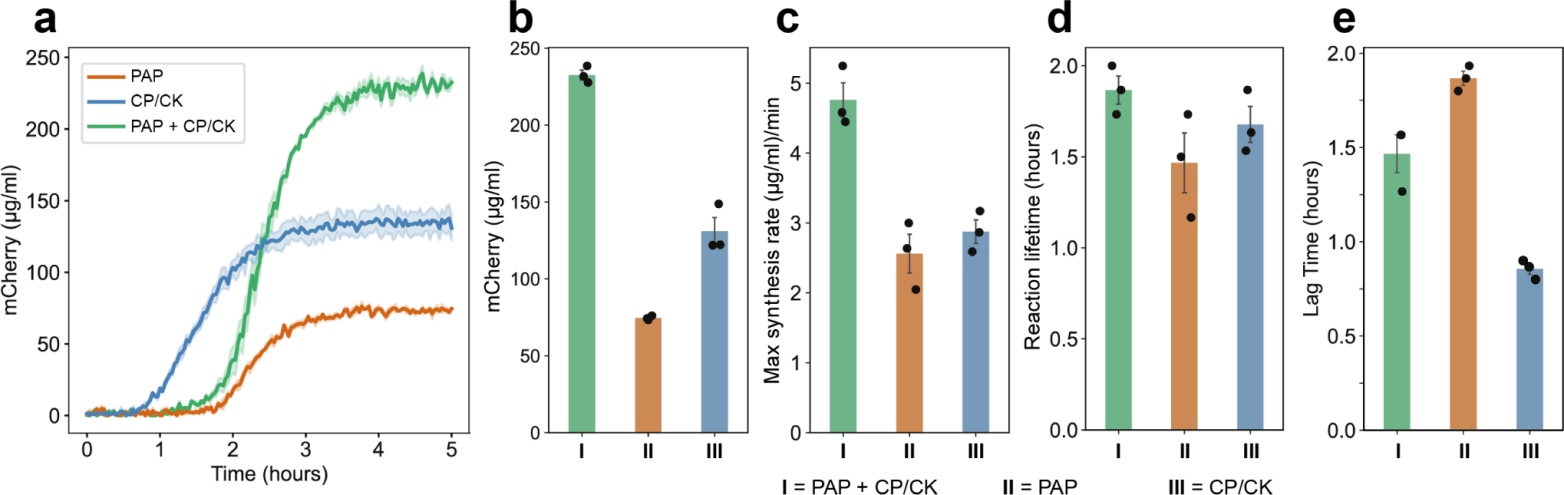
Combination of the PAP and CP/CK pathways significantly increased both the rate of protein synthesis and the final protein yield. (a) Protein expression kinetics of PURE reactions utilizing different ATP regeneration pathways are shown. (b) Final protein synthesis yields after 5 h. PURE reactions utilizing the CP/CK system gave a higher protein yield compared to those using optimized PAP; however, the combined PAP + CP/CK system exceeds either system alone. (c) Maximal protein synthesis rates are shown for each system. Similar to the yield, the combined PAP + CP/CK system exhibited the highest maximal protein synthesis rates. (d) The reaction lifetime remained approximately constant in all three cases. (e) Reactions containing PAP exhibit increased lag time compared to the CP/CK reaction. All experiments were performed in triplicates. Data are shown as mean ± s.e. (*n* = 3).

The DOE-optimized pathway resulted in a yield of 74.5 ± 0.8 μg/mL of mCherry. Compared to the CP/CK system alone, which produces 130.9 ± 8.0 μg/mL, the PAP system does not serve as an effective replacement (Figure 4a, orange and blue curves); however, we hypothesized that the two pathways in combination might achieve a higher total yield.

To accomplish this, the final reaction mixture was supplemented with both creatine phosphate and the PAP components (Table S5). This setup endowed the PURE system with two ATP regeneration pathways, CP/CK and PAP, each utilizing different substrates. The combination of both systems led to a significant increase in protein yield up to 232.6 ± 3.2 μg/mL of mCherry, marking a 77.6% enhancement compared to using only the CP/CK system, and 212% increment compared to using only PAP (Figure 4a,b). Concurrently with the increased yield, the combined system also shows an increase in the maximal rate of protein synthesis (Figure 4c).

### 2.5 PAP-Powered Reactions Exhibit Increased Lag Time for Fluorescent Protein Production

We additionally assessed the kinetics of protein expression by analyzing two time scales associated with the reaction. The first is the lag time, defined as the time interval from the start of the reaction until the first appearance of a fluorescence signal: this is the minimal time for synthesis and maturation of the first fluorescent proteins. The second measure is the reaction lifetime, which we defined as the time interval between the first appearance of signal until the saturation of the fluorescence at its maximal value. This is equal to the time interval over which protein synthesis remains active. Details of this analysis are given in Figure S14.

Our original hypothesis that the reaction lifetime would be increased under the PAP system was not proven, as this interval remained roughly constant between the CP/CK and PAP reactions (Figure 4d). However, we observe that PAP-powered reactions exhibit an increased lag time before the first appearance of mCherry fluorescence (∼1.5 h), compared to CP/CK-powered reactions (<1 h) (Figure 4e). This could be due to a number of reasons, including a delayed start to transcription and/or translation, as well as a delay in the maturation of the fluorescent protein.

Further analysis of the DOE data set revealed that pyruvate concentration was correlated with lag time (Figure S15): the greater the initial concentration of pyruvate, the longer the delay. Our first hypothesis was that an increase in pyruvate would result in increased H_2_O_2_ production, which would be inhibitory to CFPS as demonstrated earlier. This should be compensated by the addition of additional catalase; however a titration of catalase did not reveal any effect on lag time (Figure S16).

Limited availability of acetyl phosphate, and hence ATP, can also be ruled out as an explanation for the increased lag time, as under that hypothesis the lag should decrease with increasing pyruvate. Additionally, the increased lag is present in the combined CP/CK + PAP system, where ATP availability is assured from the CP/CK pathway. Thus, it is more likely that it is the fluorescent protein maturation, rather than CFPS, which is responsible for the increased lag.

Since the pyruvate oxidase reaction is oxygen-dependent, our remaining hypothesis therefore is that a lowered concentration of dissolved oxygen brought about by Pox5 activity could delay mCherry maturation.^45^ This anaerobic state would be maintained as long as pyruvate is available, but would cease as soon as pyruvate runs out. Hence increasing initial pyruvate concentrations would increase the lag time. To test whether protein synthesis could be observed at times earlier than the onset of the mCherry signal, we expressed the alternative reporter nanoluciferase (Figure S17). Although this reaction is also oxygen-dependent, we assumed that agitation due to addition of the furimazine substrate would sufficiently reoxygenate the system. Using this approach we observed that although nanoluciferase was detectable after 1 h of reaction, the lag associated with PAP systems remained: most notably, the PAP + CP/CK system again exhibited delayed protein synthesis compared to the CP/CK system alone. The cause of this delay thus remains inconclusive.

### 2.6 PAP Can Power the Commercial PURExpress System

**Figure 5:**
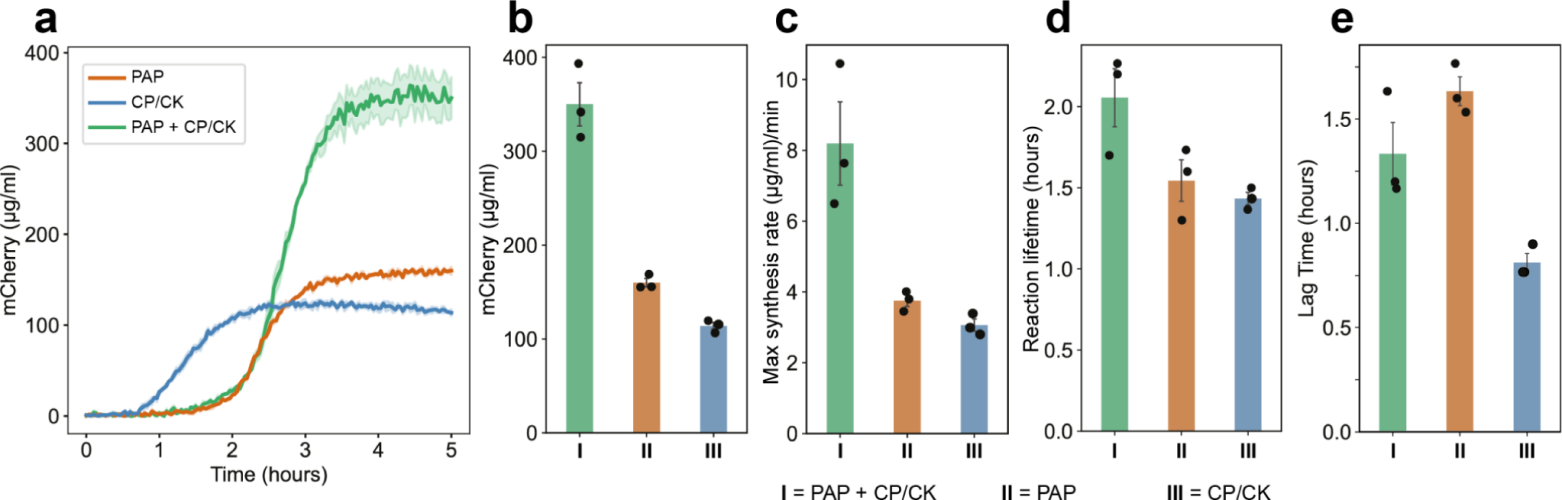
PAP functions as an ATP regeneration component in the commercial PURExpress system. Only the “Factor Mix” from the PURExpress Δ Ribosome Kit was used in the experiments as it contained all the PURE proteins. All other reaction components were homemade, including ribosomes, energy solution, mCherry DNA template, and other required additives. (a) Protein expression kinetics of “Factor Mix” reactions utilizing different ATP regeneration pathways are shown. (b) Final protein synthesis yields after 5 h are shown. “Factor Mix” reactions utilizing optimized PAP provided a higher protein yield compared to those using CP/CK. Similar to previous experiments, the combined system gave the highest yield. (c) “Factor Mix” reactions utilizing both optimized PAP and CP/CK achieved the highest protein synthesis rates, compared to each pathway alone. (d) The reaction lifetime of the combined system is marginally greater than for the individual systems. (e) Reactions containing PAP exhibit increased lag time compared to the CP/CK reaction. All experiments were performed in triplicates. Data are shown as mean ± s.e. (*n* = 3).

Finally, we assessed the reproducibility of the PAP across different batches of PURE produced in-house, as well as the commercially available PURExpress system. The pathway exhibited consistent performance across different batches of homemade PURE, yielding comparable final protein yields (Figure S18). To test PAP as an ATP regeneration component in a commercial PURE system, the PURExpress ΔRibosome Kit from New England Biolabs was used. The experiments were conducted using only the “Factor Mix” from the kit as it contained all the PURE proteins. All other reaction components were homemade, including ribosomes, energy solution, mCherry DNA template, and other required additives. Upon addition to PURExpress without creatine phosphate, PAP by itself resulted in increased protein yield (159.9 ± 4.5 μg/mL), surpassing the protein yield under the CP/CK system (113.7 ± 3.8 μg/mL). Furthermore, combining PAP with the CP/CK system in PURExpress resulted in protein yields exceeding 350.0 μg/mL, showing 207.8% enhancement compared to PURExpress utilizing only the CP/CK component, and 118.9% increment compared to only using the PAP (Figure 5a,b). Broadly similar trends to the in-house PURE data were observed with synthesis rates and lag times, although the reaction lifetime appeared marginally increased under the dual energy system (Figure 5c–e).

## 3 Discussion

In this work, we demonstrated that parallel energy regeneration systems can be straightforwardly integrated with PURE. While the original CP/CK system regenerates sufficient ATP to power protein synthesis, it has been hypothesized to be self-limiting due to inorganic phosphate accumulation.^12^ We tested a pathway which regenerates ATP concurrently with phosphate recycling, as originally proposed by Kim and Swartz.^26^ Since the pathway now relies on phosphate as a substrate, our initial observation that the pathway resulted in low synthesis yields was unsurprising, as the only source of inorganic phosphate came from ATP hydrolysis.

What was surprising however was the finding that 20 mM of phosphate buffer can activate the pathway, as such phosphate concentrations were originally suggested to be inhibitory to the PURE system in particular. Li et al. measured up to 20 mM of phosphate accumulation in PURE, and additionally showed that replenishing the reaction at later times with magnesium acetate increased CFPS activity, which they attributed to the mitigation of magnesium sequestration.^12^ However, our finding that 20 mM of phosphate buffer does not inhibit PURE reactions is at odds with the earlier results.

As is well-known, the protonation state of the phosphate anion is pH dependent: at pH 7, phosphate mainly exists in the monobasic (H_2_PO_4_^–^) and dibasic forms (HPO_4_^2–^), while at pH 4.5, it is only the monobasic form that is predominant. The dissociation constant of Mg^2+^ with dibasic phosphate is estimated to be *K*_d_ ∼ 10–60 mM.^46−48^ Thus, it is possible that magnesium sequestration would only be significant at higher P_*i*_ concentrations than the 20 mM or so which accumulates in PURE.

These observations are mirrored in studies using lysate systems: supplementation of CFPS reactions with up to 10 mM phosphate is necessary to activate glucose metabolism in lysates, as demonstrated by Calhoun et al.^49^ Importantly, they find inhibitory concentrations of dibasic phosphate are high—up to 50 mM.

The combination of multiple ATP regeneration pathways has been demonstrated before in lysates, for example with combined CP/CK and glucose metabolism.^17^ In our work we observe that the combination of PAP and CP/CK leads to roughly an additive increase in both final protein yield and maximum synthesis rate. This suggests that the pathways are independently contributing to the ATP regeneration, possibly by maintaining a higher steady-state ATP level throughout the CFPS process. Time-resolved measurements of ATP level throughout the reactions could verify this hypothesis.

In light of these findings, we conclude that while PAP functions effectively as an ATP regeneration pathway, the contribution of its phosphate recycling mechanism toward increasing reaction yield is unlikely to be significant under the conditions tested. However, PAP may enable the operation of multiple pathways whose combined effects would otherwise result in high (>50 mM) levels of phosphate accumulation, and we plan to test these conditions in future work.

Our leading hypothesis is that the inhibition observed upon addition of 20 mM monobasic phosphate is due to lowered pH rather than magnesium sequestration: it is well-known that lowered pH inhibits CFPS.^49−52^ To support this, we measured the initial pH of reactions supplemented with either monobasic phosphate or phosphate buffer. We found that increasing concentrations of monobasic phosphate lowered the pH of the reactions from 6.95 to 6.54. In contrast, increasing concentrations of phosphate buffer had negligible effects on the pH of the reactions (Figure S19).

Finally, while PAP demonstrates the feasibility of introducing a parallel, independent ATP regeneration system in PURE, there are a few weaknesses associated with the design. The oxygen requirement of pyruvate oxidase imposes limitations on scaling up the reactions beyond ∼50–100 μL. The buildup of acetate eventually lowers the pH of the reactions, and can be a limiting factor. A potential alternative pathway to explore is based on pyruvate dehydrogenase and phosphate acetyltransferase, which could generate acetyl phosphate from pyruvate, using coenzyme-A and NAD^+^ as cofactors.

Ultimately, such batch-mode reactions will inevitably reach equilibrium, and attempts to mitigate inhibition due to byproduct buildup, whether it is phosphate or acetate, can only prolong but not prevent this equilibration. Maintaining continuous activity continuously requires active byproduct removal through alternative reaction formats e.g., continuous exchange/flow reactors, or compartmentalization and controlled transport across the reaction barrier.^53,54^

## 4 Conclusion

In conclusion, we have demonstrated the construction of an ATP regeneration system based on pyruvate oxidase, acetate kinase, and catalase, and its integration into the PURE cell-free protein synthesis system. This PAP pathway utilizes pyruvate and phosphate as substrates, generating an acetyl phosphate intermediate that rephosphorylates ATP in situ. The pathway itself can function independently, producing up to 74.5 ± 0.8 μg/mL of mCherry, or it could be combined with the existing creatine phosphate/creatine kinase system in PURE to produce 232.6 ± 3.2 μg/mL of mCherry. This behavior is reproducible across multiple batches of homemade PURE, and generalizable to the commercial PURExpress system. This demonstration indicates the relative ease and flexibility of constructing parallel metabolic pathways in PURE, which should enable future applications in cell-free protein synthesis and the construction of synthetic cells.

## 5 Methods

Brief descriptions of methods are given here; for fully detailed procedures please see the “Experimental Details” section in the Supporting Information.

### 5.1 Materials

All materials, including chemicals and reagents used in this study, are listed in Table S6, complete with their respective supplier catalog numbers.

### 5.2 *E. coli* Strains and Plasmids

*E. coli* TOP10 was used for plasmid maxiprep, *E. coli* DH5α was used for plasmid maintenance, and *E. coli* BL21(DE3) was used for protein expression and ribosome purification (Table S7). Plasmids encoding PURE proteins used in this study were gifts from Sebastian Maerkl and Takuya Ueda (Addgene plasmids #124103–124138), except pET21a-MTF-6xHis. Genes coding for MTF-6xHis, Pox5–6xHis, KatE-6xHis, and AckA-6xHis were obtained as linear DNA fragments (gBlocks, IDT) (Table S8), cloned into a pET21a vector using the Gibson Assembly Cloning Kit (NEB) according to the manufacturer’s protocol,^55^ and then transformed into *E. coli* BL21(DE3) and DH5α cells. The gene coding for mCherry-6xHis was cloned into a T7p14 vector, and then transformed into *E. coli* TOP10 and BL21(DE3) cells. A list of PURE proteins along with their corresponding vector, gene, and strain are provided in Table S9. The primers used in this study are provided in Table S10. Plasmid maps and amino acid sequences of all expressed proteins are given in Tables S11 and S12.

### 5.3 PURE Proteins Preparation

The PURE proteins were prepared using a modified “OnePot” protocol based on established procedures.^10^ Briefly, strains for the PURE system were grown from standardized liquid glycerol stocks to an optical density of OD600 = 2–3, followed by coinoculation of all 36 strains into 500 mL of LB media with ampicillin. Cultures were incubated at 37 °C, with shaking at 220 rpm until OD600 = 0.2–0.3, at which point protein expression was induced using a final concentration of 0.1 mM IPTG. Following a 3-h expression period, cells were harvested and washed with PBS and flash frozen in liquid nitrogen. The cell pellets were thawed and resuspended in a lysis buffer, and lysed by sonication. Lysates were clarified at 15,923g and incubated with Ni-NTA resin for 3 h at 4 °C. Protein purification involved a single 5 mM imidazole wash step followed by elution with 450 mM imidazole. Purified proteins were dialyzed, concentrated, and adjusted to a final concentration of 12.5 mg/mL in 30% glycerol buffer. The protein solution was aliquoted and stored at −80 °C until use. PUREΔCK systems were created in the same way but with the omission of the creatine kinase strain in the main culture.

### 5.4 Crude Ribosome Preparation

Crude 70S ribosomes used in the CFPS reactions were purified using high-speed zonal centrifugation as described previously.^56^ Ribosomes were purified from *E. coli* BL21(DE3) cell cultures harvested at an optical density of OD600 = 0.6. Briefly, a starter culture was inoculated from a glycerol stock and incubated, with a parallel sample to test for media contamination. The culture was then scaled up to 4 × 750 ml through successive stages, with optical density monitoring to guide the growth to the desired cell density. Following growth, cells were cooled, harvested by centrifugation, and washed in PBS to remove media. Cell lysis was achieved via sonication, and the lysates were clarified through an initial centrifugation at 30,000*g* for 1 h at 4 °C. Clarified lysate was subsequently ultracentrifuged at 100,000*g* for 4 h at 4 °C to obtain crude ribosome pellets. These pellets were then processed through several resuspension and centrifugation steps using high salt buffer to further purify the ribosomes. Finally, the ribosome concentration was determined using Nanodrop spectrophotometry, and the ribosome solutions were aliquoted and stored at −80 °C until use.

### 5.5 Buffers Used for Protein and Ribosome Purification

Buffers used for protein and ribosome purification are listed in Tables S13 and S14. All buffers were filter-sterilized using bottle-top filters with a 0.2 μm PES membrane, and stored at 4 °C until use. Reducing agent TCEP (for proteins) or DTT (for ribosomes) was added immediately before use.

### 5.6 Plasmid Maxiprep of T7p14-mCherry-6xHis

Plasmid DNA was purified using a modified ZymoPURE II Plasmid Maxiprep kit protocol, adjusted to process the equivalent of three standard reactions to accommodate higher plasmid quantities. The protocol commenced with inoculation of a single colony or glycerol stock into LB media with ampicillin, followed by 500 mL overnight culture. Postculture, cells were harvested and subjected to a lysis-clearing-neutralization sequence using ZymoPURE reagents P1, P2, and P3. Following lysis, cell debris was removed using ZymoPURE Syringe Filter-X units, ensuring critical attention to avoid sample loss and ensure the recovery of 70–100 mL of cleared lysate. The lysate was then mixed with ZymoPURE Binding Buffer, and the DNA-bound mixture was processed through Zymo-Spin V-PX Column assemblies with subsequent wash steps to remove contaminants. DNA was eluted in 500 μL of Nuclease-Free Water, preheated to 55 °C. This elution was further purified for endotoxins using EndoZero Spin-Columns, with elutions combined for final DNA concentration and purity assessment via Nanodrop spectrophotometry, targeting a yield of around 900 ng/μL.

### 5.7 Energy Solution Preparation

The energy solution for PURE reactions was prepared as previously described,^10^ with several modifications. A 4x energy solution, excluding creatine phosphate, magnesium glutamate, and potassium glutamate, was prepared. This solution contained 200 mM HEPES, 8 mM ATP, 8 mM GTP, 4 mM CTP, 4 mM UTP, 14 mg/mL tRNA, 4 mM TCEP, 0.08 mM folinic acid, 8 mM spermidine, and 1.2 mM of each amino acid (with the exception of leucine, which was at 1 mM).^10^ For more information, refer to Table S15.

### 5.8 Reaction Setup for Cell-free Protein Synthesis

PURE reactions utilizing only the CP/CK ATP regeneration system were prepared in a 50 μL master mix containing 12.5 μL of 4x Energy Solution, 20 mM creatine phosphate, 2.4 mg/mL PURE Protein solution, 2.275 μM ribosome solution, 11.8 mM Mg-glutamate, 100 mM K-glutamate, 2% PEG 8K, and 10 nM mCherry plasmid DNA template. Other reaction components such as phosphate (if added) varied according to the reaction requirement. PURE reactions utilizing both CP/CK and PAP systems were prepared in a 50 μL master mix containing 12.5 μL of 4x Energy Solution, 20 mM creatine phosphate, 2.4 mg/mL PURE Protein solution, 2.275 μM ribosome solution, 12.5 mM Mg-glutamate, 100 mM K-glutamate, 2% PEG 8K, 27.5 mM K-phosphate buffer (pH 7), 27.5 mM pyruvate, 3.03 μM Pox5, 13.89 μM AckA, 2.38 μM KatE, 2 mM TPP, 0.2 mM FAD and 10 nM mCherry plasmid DNA template. PURE reactions utilizing only the PAP system were prepared in a 50 μL master mix containing 12.5 μL of 4x Energy Solution, 2.4 mg/mL PURE Protein solution, 2.275 μM ribosome solution, 100 mM K-glutamate, 2% PEG 8K, 13.89 μM AckA, 2 mM TPP, 0.2 mM FAD, and 10 nM mCherry DNA template. All other reaction components, including enzymes, substrates, etc. varied according to the reaction setup. Detailed reaction setups are provided in Table S5. PURExpress reactions (50 μL master mix) utilizing CP/CK, or PAP, or both components were set up in the same way as mentioned above, except the PURE protein solution was replaced with 6 μL PURExpress Factor Mix. PURExpress control reaction (50 μL master mix) contained 20 μL Solution A, 6 μL Factor Mix, 9 μL NEB ribosomes, and 10 nM mCherry DNA template (Figure S20). All the reaction master mixes were prepared on ice, mixed using pipetting and split into three 15 μL reactions in a 384-well plate (Greiner 384 μClear Black). The protein synthesis kinetics of reactions were then measured on a BioTek Synergy H1 plate reader (excitation, 579 nm; emission, 616 nm; gain 50; bottom optics; double orbital 2 s shaking before every read), taking fluorescence readings every 2 min for 5 h. Relative fluorescence units were converted to physical concentration units of active fluorescent protein using a calibration curve (Figure S21). Technical triplicates were taken of all reactions. Biological replicates of enzymes, PURE, and ribosomes are detailed in Table S16.

### 5.9 Cloning and Purification of Enzymes

Genes encoding the three pathway enzymes, each with a C-terminal 6x His-tag, were obtained as gBlocks from Integrated DNA Technologies (IDT) and individually cloned into the pET21a expression vector using the Gibson Assembly Cloning Kit (NEB) according to the manufacturer’s protocol. Primers used to generate the Gibson Assembly fragments for each gene are listed in Table S10. The assembled constructs were transformed into DH5α cells, and positive clones were selected on LB agar plates containing ampicillin. Subsequently, the assembled plasmids were miniprepped from the DH5α cells using the ZymoPURE Plasmid Miniprep Kit (Zymo Research), according to the manufacturer’s instructions, and transformed into BL21(DE3) cells for protein expression and purification. Protein expression and purification of the pathway enzymes were carried out according to the PURE Proteins Preparation Protocol detailed above, with several modifications. The enzyme strains were cultured overnight in LB-Amp media from their respective glycerol stocks, followed by inoculation into 500 mL of LB-Amp media. After protein purification, each enzyme was dialyzed, concentrated, and stored in 30% glycerol buffer at −80 °C for future use. The Pox5 enzyme required a modified expression protocol to address protein insolubility issues; protein expression was conducted at 15 °C for 15 h.

### 5.10 Design and Analysis of DOE

The circumscribed central composite design (cCCD) is a symmetrical design consisting of: a center point at the design space’s center, 8 corner points forming a box around the center, representing a 2-level full factorial design, and 6 axial points set at the minimum and maximum levels for each factor, positioned ±1.68 times the distance from the center point to each corner point.^40^ 19 conditions were set up, comprising 15 from the cCCD, and 4 from a holdout test data set identified through Latin hypercube sampling within the bounds of the design space, to validate the model.^40^ Each of the reaction conditions were compiled in triplicate, except for the center point which had six replicates, and the end point readings were taken at 5 h. Linear regression was used for model fitting. Design generation, modeling and plotting was performed in Python.

## Supporting Information Available

Additional experimental data for enzyme purification and activity assays, negative/positive controls, phosphate/pH experiments, calibrations, and DOE analysis; full experimental details and conditions, ODE model formulation and parameters, data sets, and list of materials (PDF)
